# Rural patients’ experiences with diagnosis and treatment of endometrial cancer

**DOI:** 10.1111/jrh.70065

**Published:** 2025-08-22

**Authors:** Victoria M. Petermann, Brianna D. Taffe, Blen M. Biru, Jennifer Leeman, Ashley Leak Bryant, Benjamin B. Albright, Stephanie B. Wheeler, Victoria L. Bae‐Jump, Lanneau Grainger, Lisa P. Spees

**Affiliations:** ^1^ School of Nursing University of Virginia Charlottesville Virginia USA; ^2^ Department of Epidemiology, Gillings School of Global Public Health University of North Carolina Chapel Hill North Carolina USA; ^3^ Center for Health Equity Research University of North Carolina Chapel Hill North Carolina USA; ^4^ School of Nursing University of North Carolina Chapel Hill North Carolina USA; ^5^ Division of Gynecologic Oncology, Department of Obstetrics and Gynecology University of North Carolina Chapel Hill North Carolina USA; ^6^ Department of Health Policy and Management, Gillings School of Global Public Health University of North Carolina Chapel Hill North Carolina USA; ^7^ Lineberger Comprehensive Cancer Center University of North Carolina Chapel Hill North Carolina USA; ^8^ Division of Gynecologic Oncology, Brody School of Medicine East Carolina University Greenville North Carolina USA; ^9^ Division of Pharmaceutical Outcomes and Policy, Eshelman School of Pharmacy University of North Carolina Chapel Hill North Carolina USA

**Keywords:** access to treatment, barriers to diagnosis, endometrial cancer, multilevel barriers, rural cancer patients

## Abstract

**Background:**

Rural endometrial cancer (EC) patients are less likely to receive lymph node evaluation, high‐quality surgical care, and adjuvant therapy compared to urban patients. Developing interventions to effectively address barriers to quality care requires understanding patient experiences across the cancer care continuum. Our objective was to understand the diagnostic and treatment experiences of rural EC patients.

**Methods:**

We conducted semistructured interviews with 23 participants (22 patients, one caregiver) from rural counties in North Carolina. We developed a semistructured interview guide to examine the experiences of patients during diagnosis and treatment. Initial codes were derived from a multilevel conceptual framework of rural cancer control, and transcribed interviews were analyzed using thematic analysis.

**Results:**

We identified six themes reflecting determinants of diagnosis and seven themes for treatment of EC for rural patients. Provider knowledge of EC symptoms, patient symptom normalization, and fear were all discussed as major factors impacting delays in EC diagnosis. Participants noted that social networks influenced them to seek care for symptoms they did not otherwise see as concerning. During treatment, participants experienced financial burdens, and many reported significant challenges traveling to treatment. Social networks were critical for financial support and transportation to and from treatment. Personal health care experiences and community perceptions about rural cancer care also influenced decisions about where to seek gynecologic cancer treatment.

**Conclusions:**

This study highlights the need to improve rural provider adherence to guidelines for EC detection, increase symptom knowledge among rural communities, and implement comprehensive assessments of unmet needs of rural patients during treatment.

## INTRODUCTION

Endometrial cancer (EC) is the most common gynecologic malignancy diagnosed in the United States. Roughly 66,000 cases are diagnosed in the United States each year, and the incidence is projected to increase from 27 cases per 100,000 women in 2010 to 42 cases per 100,000 women in 2030.^1^ Prompt evaluation and referral to gynecologic oncology services is critical for early diagnosis and ensuring high‐quality care. Yet, individuals diagnosed with EC residing in rural settings experience more delays in care.^2^ During treatment, consistent evidence demonstrates that rural EC patients receive poorer quality care,^2^ including a lower likelihood of receiving lymph node evaluation,^3^, ^4^ high‐quality surgical care,^5^ and adjuvant therapy,^2^, ^6^ compared to their urban counterparts.

Numerous factors may explain why individuals in rural areas are at risk for inequities in diagnosis and treatment for EC. Factors that may impact prompt diagnosis include inadequate access to primary or gynecologic care leading to delays or discontinuity in care,^7^ provider recognition of EC symptoms,^8^, ^9^ and lack of providers comfortable performing guideline‐adherent procedures, such as an endometrial biopsy.^10^ In addition to limited health care access, socioeconomic inequities, including costs^11^ and lower educational attainment,^8^, ^12^ also have potential to negatively impact the diagnostic experience of rural EC patients. Cancer treatment can also be costly, create high travel burdens, and be complex to manage, all of which may impact outcomes for those living in rural settings.^13^, ^14^ Treatment by a gynecologic oncologist is associated with better outcomes for patients with gynecologic malignancies.^6^ However, EC patients in rural areas are more likely to lack access to gynecologic‐oncology services, resulting in significant barriers to care and substantial reliance on financial and social support from caregivers.^14^, ^15^

Patient perspectives can provide valuable information for understanding how barriers and facilitators to care operate. While there have been qualitative studies examining the long‐distance travel for treatment for patients diagnosed with gynecologic malignancies^14^ and survivorship care for rural EC patients,^16^ no studies have qualitatively examined the diagnosis or treatment experiences of rural patients with EC. Rural areas are defined by more than distance, and understanding the ways in which physical distance and culture impact rural EC patients’ experiences across the cancer care continuum is critical. We contribute to these previous qualitative studies by exploring the rural patients’ experience of determinants of access to care during diagnosis and treatment of EC.

## METHODS

This study uses data collected from two successive studies that interviewed patients to identify determinants of access to care for rural EC patients. These studies were approved by the University of North Carolina Institutional Review Board (UNC‐IRB #23–0007 & # 22–3204).

### Setting and participants

Patients were recruited through gynecology oncology clinics of two different health care systems in North Carolina between January 2021 and February 2023. Participants in the first study were recruited solely from one health care system, and participants in the second study were recruited from both health care systems. Eligible patients were identified through a review of electronic patient records by B.B. or V.M.P. at one health system. At the second health system, eligible patients were identified by clinic staff, and patient data were shared with the research team. Patients were deemed eligible for recruitment if the patient (1) had a diagnosis of EC, (2) completed treatment ≤3 years from the study start date, (3) lived in a rural county (codes 4–9 based on the 2013 Rural‐Urban Continuum Codes),^17^ and (4) spoke and understood English. In the first study, we limited recruitment to individuals who (1) were at least 50 years old and (2) had received adjuvant therapy during EC treatment. We also used a stratified sampling approach to equitably recruit individuals residing in rural counties with high proportions of Black individuals (defined as counties above the 2020 state county average of Black individuals in the state [22%]). We used these criteria because individuals who are older, live in rural areas with high populations of Black individuals, or undergo prolonged treatment often experience more barriers to care. Due to recruitment challenges, we relaxed our criteria during the second study and no longer prioritized patients based on age, county racial composition, or type of treatment. Interviewees were compensated with a $25 Visa gift card in the first study and a $50 Visa, Amazon, or Walmart gift card in the second study for their participation.

### Interview guide

We developed questions for the semistructured interview guided by the Andersen and Aday Behavioral Model of Access to Care.^18^, ^19^ The interview guide consisted of separate sections on diagnosis and treatment experiences, both of which included questions crafted to probe the multilevel factors that may have impacted participants’ experiences accessing care (Appendix S1). Following the analysis of interviews from the first study, the interview guide was modified to ask additional questions about how the health system could improve the treatment process and access to supportive care resources.

### Recruitment and data collection

For patients meeting inclusion criteria, a recruitment letter with information about the study was sent to the patient. A study team member (V.M., B.B.) would then follow up with the participant by phone to recruit the participant and either conduct the interview or schedule it for another time. Semistructured interviews were conducted over the phone or through an audio‐only video‐conferencing platform and lasted 45–60 min. Interviews were conducted by V.M.P. (who was a doctoral student and postdoctoral fellow during the time of these studies) and B.B. (a project manager with a master's in public health). Both V.M.P. and B.B. received training and had prior research experience conducting qualitative interviews. Verbal consent was obtained prior to starting the interview, and the interviewers shared the purpose and goals of the study with participants as part of the consent process. For patients who met the inclusion criteria but were unable to participate in an interview, we conducted interviews with a caregiver. Basic demographic and clinical information was obtained from the participant's chart. In the initial study, we recorded demographic data from the patient's chart. In the second study, we altered our approach and collected this information at the end of interviews, as self‐report is a more rigorous approach to documenting race/ethnicity. All interviews were audio recorded and transcribed verbatim. Interview transcriptions and data about participants were de‐identified and stored on a secured, password‐protected server. Sample size was determined by both methodological and practical factors. We aimed to collect interviews from 20 participants to ensure the collection of rich data while having the resources to meaningfully analyze and interpret the data. We ceased recruitment when, after many team discussions during the analytic process, we determined there to be informational redundancy, and no new topics were being addressed in the interviews.

### Data analysis

A descriptive, thematic approach was used to analyze participant interviews. Thematic analysis is a method of descriptive qualitative analysis that develops patterns of meaning (or themes) across a dataset.^20^, ^21^ This process of analysis follows roughly six iterative processes: (1) familiarization with the data, (2) generating initial codes, (3) coding data, (4) examining coded data for themes, (5) reviewing themes for fit with the data, and (6) defining and naming themes.^20^, ^21^ The initial codebook was created guided by two multilevel socioecological models of the determinants of rural cancer care^22^, ^23^ and an initial reading of the transcripts. Two members of the team independently coded each of the transcripts (V.M.P., L.P.S., B.B., or B.D.T.) using Dedoose (Version 9.2.6 [2024], Los Angeles). Additional inductive codes were added throughout the coding process as needed. Coding disputes were discussed and resolved by consensus among coders, and meeting notes were used as an audit trail for analytic decisions. The coded data were then reviewed to identify the initial themes present in the data, which were then verified among all coders. Findings were reviewed by clinicians and research experts in gynecologic oncology and rural cancer care.

## RESULTS

**FIGURE 1:**
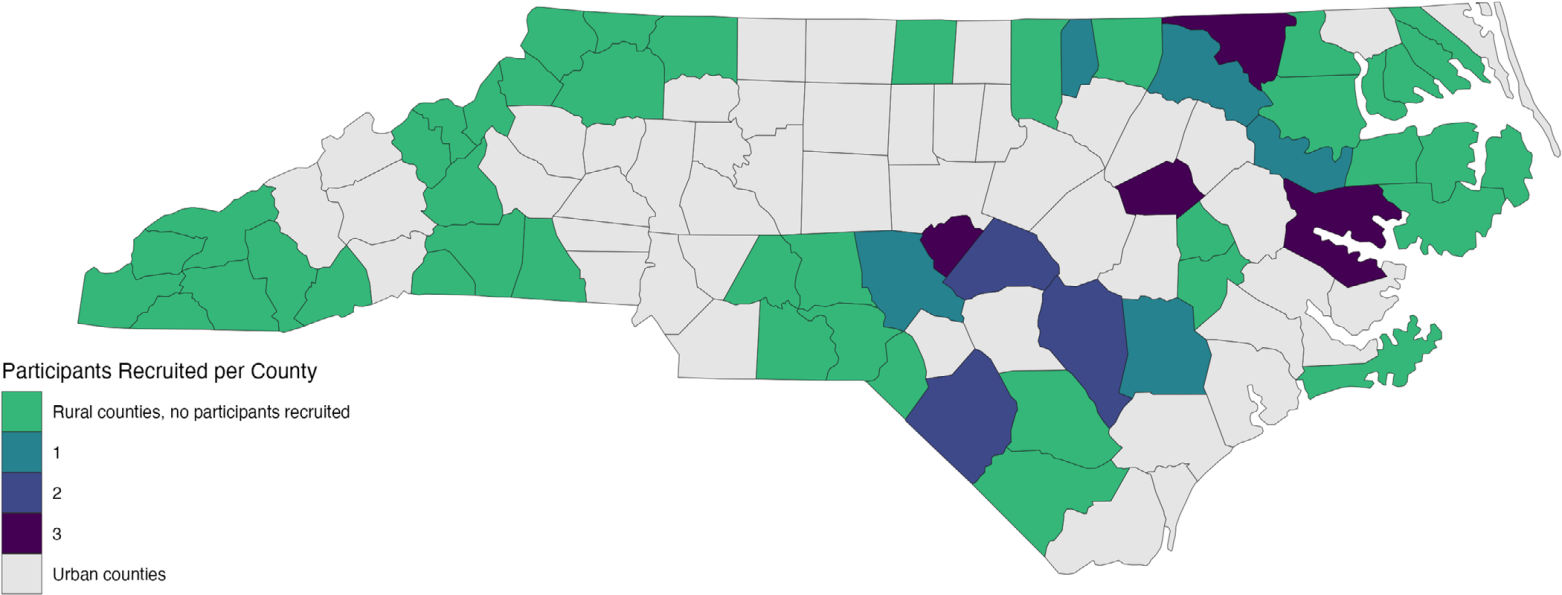
Number of Participants Recruited from Rural Counties in North Carolina

A total of 90 individuals were contacted for interviews, and 23 agreed to participate. Twenty‐two patients from rural counties who had completed treatment for EC and one caregiver whose parent had completed EC treatment participated in interviews (Table 1). Participants were recruited from 12 counties, primarily in the central and eastern regions of the state (Figure 1). Within two phases of care (diagnosis and treatment), we identified 13 themes reflecting multilevel determinants of access to care (Table 2, 3).

**TABLE 1:** Patient demographics.

	*N* = 23
Patient characteristic	*n*
Age (range)	58 (38, 71)
Race	
Black	7
White	13
American Indian	1
Multiracial	1
County demographic	
>22% Black	17
<22% Black	6
Stage	
I	14
II	2
III	2
IV	1
Unknown	4
Adjuvant therapy	
Radiation	15
Chemotherapy	10

**TABLE 2:** Diagnosis experience themes.

Theme	Exemplar quote
Provider capacity^a^	“I went to my first visit…it took a while to get, actually get in to the clinic.”—P08
	“I didn't wait that long [to go see my doctor]. And then after that, she referred me to an oncologist‐gynecologist, and it wasn't late either. I mean it was fast.”—P12
Symptom recognition by providers	“Well, I've been bleeding for over two years and then because of my health problems already, [my health care provider] didn't choose to do anything then until it got so bad, the bleeding got so bad, that I had to go and they finally did a biopsy.”—P11
Symptom normalization by patients	“I just got up one morning and went to the bathroom and there was blood. And I had been in menopause for seven or eight years at that point. And I didn't think much about it at first. I just thought maybe I scratched myself. It's nothing. But it lasted. It continued for several days and I had had it once before, maybe three or four months before that.”—P19
Fear and care avoidance	“I just kept putting it off. I hated to go. It's a personal experience to me. I'm just not used to going to a doctor to get a pap smear on a regular basis. So it was a little challenging, that part of it.”—P23
Encouragement to seek care	“My daughter was going to this doctor at the time. She was pregnant, she [was] worried and she told me about her. She said, “Mom, come with me to this doctor.” So, I went and they set me up an appointment and [the doctor] examined me.”—P09
Ease of gynecologic oncology referral process	“[My OB/GYN] referred me to an oncologist gynecologist and it wasn't late either. I mean it was fast. Everything was pretty fast.”—P12

^a^ In these themes, participants reported divergent experiences: some experienced a barrier, while others did not. As such, we have provided multiple quotes to show the range of experiences patients had with regard to determinants of access to care.

**TABLE 3:** Treatment experience themes.

Theme	Exemplar quote
Physical and financial burdens of distance to care	“The only bad thing that I had was the trip, the long trip. It just…Going out there and then went through all the procedures and then having ride back home, that's an all day thing and it just wore me out.”—P04
Financial burden resulting from insurance coverage quality^a^	“I have to pay, like, copays, but they're just small. They're like $30 here, $30 there”—P08
	“I'm still paying. So since then, I start paying $168 a month. Yeah. $168 and some change so that I've been paying for two years and I still have a balance. I still have to pay more. So yeah. I owe like $3,000 maybe.”—P12
Financial and other instrumental support from social network	“I have another brother that lives behind me so he comes up. If I need anything and I can't do it, I can get him to do it. Or if I need something from the grocery store and I don't feel like going it, then he'll go down there for me.”—P23
Referral to supportive services	“I don't recollect that that they offered me anything like that even though I was doing that time with them.”—P20
Insurance mediated delays in care^a^	“They're very good there at his office about getting your right in and seen. I've never had to wait very long or anything like that or be re‐scheduled or anything.”—P17
	“My insurance [didn't] want the neurologist [close to my town]. They wanted a neurologist [elsewhere], for some reason. It was kinda crazy.”—P08
Quality of communication with providers	“The [gynecologic‐oncologist] at the hospital was wonderful. I can't tell you his name, I would have to look it up again, but he was wonderful. He came in, was very calm and told me I could ask anything…and the nurses, the same thing.”—P17
Trust in rural health care and personal experiences driving care decisions^a^	“I just don't trust the doctors that are [at the local hospital] to be as knowledgeable.”—P03
	“I certainly have got friends here that have been through [cancer] that are doing [their treatment] locally. So, I feel like it would've been okay, but I stuck with [where I had been before].”—P11

^a^ In these themes, participants reported divergent experiences: some experienced a barrier, while others did not. As such, we have provided multiple quotes to show the range of experiences patients had with regard to determinants of access to care.

### Diagnosis

Participants discussed determinants at the patient, social, health care team, and health system levels that affected their access to care during the EC diagnosis process (i.e., recognizing symptoms and seeking care for those symptoms).

### Provider capacity

Most participants reported few barriers to obtaining an appointment to see a provider once they had decided to seek care to address EC symptoms.

“It was maybe a few weeks. Maybe like two weeks. I mean it was fast.”—P12

However, a couple of participants noted challenges in access to gynecologic care in their community. One participant mentioned delays in scheduling an appointment with their gynecologic health practitioner because of limited provider availability, and another discussed how she lived in an area with a lack of access to obstetrician–gynecologists (OB/GYN) even though she was able to travel to get the care she needed.

“One [OB/GYN office] here and I don't know what people do. I know there's the health department, but I don't know what people do. Thank goodness I have a vehicle and I could go all the way to [city with a major health system] because it's an hour away from me‐But I don't know what people do here that don't have access to that.”—P17

### Symptom recognition by providers

A major factor influencing timely EC diagnosis was a provider's ability to recognize abnormal uterine bleeding or postmenopausal bleeding (AUB/PMB) as a key symptom of EC and to subsequently order the appropriate diagnostic tests and referrals. These providers were often described by participants as their primary care provider (PCP) and, in one instance, emergency room physicians. A few participants reported that their PCPs were able to quickly recognize the need for further gynecologic workup for patients experiencing AUB/PMB. However, several participants experienced delays.

Multiple participants were treated for other gynecologic conditions (e.g., yeast or urinary tract infections) before eventually being referred for a biopsy.

“We have a great family doctor, a regular doctor, and she had been treating me for yeast infections. And it kept going and it kept going, and it seemed like it wasn't really affecting it. I'd just get over it, and within a month or so, I'd have to have more, another antibiotic.”—P13

Another participant's provider dismissed her PMB as a “hormonal issue,” leading her to see multiple, different providers before finally seeing a “women's health specialist” who conducted an endometrial biopsy.

“They kept telling me my hormones was acting up, this and that…and all the doctors I went to, it had to be three or four doctors…and they were just coming back and forth.”—P09

Generally, once participants saw a gynecologist, they were able to quickly receive a biopsy or other diagnostic test.

“So I went to my regular doctor…my general practitioner. And I sat in his office and cried. I said, ‘You have to send me somewhere. I can't do this anymore.’ So he referred me…but first visit…[OBGYN]’s like, ‘I can't believe no one's ever done a biopsy on you before.’”—P17

### Symptom normalization by patients

Most participants reported AUB/PMB for varying lengths of time, ranging from weeks to years, before seeking care for those symptoms.

“Well, I've been bleeding for over two years and then because of my health problems already, they didn't choose to do anything then until it got so bad, the bleeding got so bad, that I had to go and they finally did a biopsy. It's gone on for two years.”—P11

Often, patients initially believed PMB was not worrisome and a normal part of menopause. For example, one participant who was postmenopausal described experiencing spotting for about a year; it was not until she experienced an instance of heavier bleeding that she decided to seek care.

“I had some postmenopausal bleeding, and I didn't think anything of it because I knew some other people. It seemed normal.”—P22

Another patient experienced intermenstrual and postcoital bleeding for almost 3 years but did not seek out care until she started experiencing systemic effects (e.g., dizziness).

### Fear and care avoidance

Even when participants reported knowing that AUB/PMB was abnormal, they mentioned fear as a major factor for not wanting their AUB/PMB evaluated. One participant reported waiting for roughly 2 years before having her PMB evaluated because she was afraid of what her symptoms may mean, while another patient avoided care because of discomfort with gynecologic exams.

“I was kind of scared at first by going to the doctor to really find out what was going on… I was just scared.”—P05

### Encouragement to seek care

PCPs played a critical role in addressing patient hesitancy. In the few instances in which a PCP recognized PMB as a symptom of EC, patients reported that the PCP addressed their hesitancy and encouraged them to pursue a diagnostic evaluation. Family members and friends also served an important catalytic role in the diagnostic process, as they would often inform participants that PMB was unusual and urge them to seek care.

“…had my daughter not said anything to me, I might not have gone to get checked. But her comment to me was, ‘Unexplained bleeding needs to be investigated no matter how small an amount.’”—P02

This was crucial for those patients who normalized their own symptoms or who had their symptoms previously dismissed by providers in prior health care visits. However, this was dependent on participants being comfortable sharing their symptoms with others. For example, a patient's caregiver mentioned the patient's reluctance to share her symptoms with close family members, which ultimately delayed the EC diagnosis.

“She kept it from us for a while. She didn't want to let us know that anything was going on with her.”—P03

### Ease of gynecologic‐oncology referral process

Once a patient received a biopsy and a cancer diagnosis, the referral process to a gynecologic oncologist was often quick. Participants estimated that the process of initial evaluation to consult with a gynecologic oncologist or undergoing surgery ranged between 2 and 6 weeks; patients frequently expressed surprise and perceived treatment initiation to be faster than they expected.

“I must say everybody was very prompt in getting me from one appointment to another as quickly as possible. I think I was at [a cancer center] at the end of that week.”—P10

### Treatment

When describing their experience receiving EC treatment, participants discussed determinants at the social, health care team, and health system levels. These determinants were primarily related to travel burden, care affordability, trust in local health care, and the interpersonal aspects of their care experience.

### Physical and financial burdens of distance to care

The amount of time participants had to travel round trip for a single appointment ranged from 1 to 6 h. Some participants discussed feeling accustomed to traveling longer distances and did not perceive travel time to be an inconvenience.

“I mean, it's like an hour and 10 minutes…it really wasn't a big deal.”—P01

Others viewed the distance as burdensome but felt that the time spent traveling was worth it for receiving high‐quality care.

Patients having to make repeated trips for adjuvant therapy (i.e., chemotherapy or radiation) also experienced high out‐of‐pocket expenses due to the cost of travel, adding to their financial burden. Two participants reported incurring additional out‐of‐pocket expenses for car rentals to drive to appointments because they did not have a car that was reliable enough to make the several trips to radiation or chemotherapy appointments.

“I have to go and rent a vehicle for him to drive me up there. So it's a money thing also getting there because I'm on a budget, because I'm retired. What I get is what I get and that's what I got to live off of.”—P23

### Financial burden resulting from insurance coverage quality

There was variability in patients’ experience of financial hardship from treatment, which patients often associated with the quality of their health insurance coverage. In particular, patients with high‐deductible insurance plans were more likely to struggle financially than patients with other types of health insurance plans. A couple of patients reported experiencing unmanageable financial burdens despite obtaining secondary health insurance. For example, one patient who was unable to continue working due to her cancer treatment also lost her home and her car and was struggling to pay her bills.

“I had to get a second insurance. But I don't know that they use all those…right now I'm, I'm stuck with so many bills that I can't go forward until I try to get them paid.”—P09

Consequently, patients largely reported using payment plans to manage these costs, and a few patients reported that they continued to struggle with meeting the monthly payments. To cope with the financial hardship from treatment, patients limited their spending in other areas of their life, obtained additional employment, or borrowed money from family. Despite the financial burdens these patients experienced, they did not identify finances as a deterrent to attending appointments or seeking care when needed. In fact, many patients were unaware of these high costs until after treatment was completed. One patient felt that the amount she was required to pay came as a surprise and felt she was not adequately informed of what the costs of care would be ahead of time.

“But for the radiation, it was 1000 dollars, and that was a big slap in the face, I wasn't prepared for that.”—P21

Several patients said they experienced no financial impact related to the cost of care; they attributed this to having insurance that covered their cancer care, and some even expressed surprise at how little they were left to pay. These patients included individuals with a variety of different insurance coverage types including Medicaid, Medicare, private insurance, and group share plans.

“I have Medicare and I have a Aetna supplement, and other than just, well I'd say less than $200, everything was covered so far, and the insurance did very well…when I saw the bill, I was so glad.”—P10

However, even patients who reported not experiencing financial hardship still discussed using payment plans to pay down the cost of care after insurance.

“My insurance is pretty good so they covered I think just about everything. I wasn't left with a whole lot. I had to pay that down a little bit at this time.”—P18

### Financial and other instrumental support from social network

Participants reported relying on caregivers or family members for financial support to cover costs from their medical bills and for transportation. To manage the high costs associated with travel to appointments, patients often relied on caregivers and their social networks to provide financial and logistical support for the increased travel burden.

“[My family was] there for me financially, because my husband's disabled and just the trips to [my appointments] all those times, it was expensive.”—P04

Rural EC patients often relied on family members to help cover the cost of travel to treatment, especially if they or their partner were unemployed, were on disability, or had a fixed income. Additional financial burdens were incurred if participants did not have reliable transportation to their appointments like for those patients who, for example, used rental car services.

In addition to financially relying on social supports to cope with the travel burdens of care, participants also relied on those supports to facilitate travel, including family, friends, acquaintances, and religious communities. In some cases, social supports incurred financial burdens (e.g., lost wages from missing work) as a result of providing transportation for patients.

“My son had to take off work, so he lost a day of pay, he is on hourly pay. So, he had to lose a whole day of pay to go and come with me [to my appointments]. That was harder for him to lose that, but he did that because he wanted to…”—P21

Patients receiving chemotherapy especially relied on transportation support due to the physical toll of treatment. However, for one participant, looking for support from family members was not always reliable. Often, she had to piece together transportation support from her broader social network, including people with whom she worked.

### Referral to supportive services

Only a few participants discussed using some supportive services, such as gas cards, to help offset their transportation costs. One participant reported being able to use a transportation program provided by her insurance plan that would drive her to and from her appointments for free. Another participant reported using temporary housing during her radiation therapy.

However, when asked about financial assistance or other instrumental supportive resources, most patients reported that they were unaware that such resources existed or that they were emotionally overwhelmed by their new cancer diagnosis and did not think to ask.

“I know they provide social work and meal tickets, but I wasn't even thinking about stuff like that during that time. The only thing I was thinking about getting that poison out of me.”—P20

Other patients reported that the resources offered were not accessible either because of the capacity of the resource (e.g., lodging resources were full when the patient desired to use them) or restrictions on how the resource could be used (e.g., transportation services could only transport the patient, not the patient *and* their caregiver).

### Insurance‐mediated delays in care

Few participants mentioned delays or wait times for care during their treatment process. However, for those who did experience delays, delays were primarily caused by insurance. One participant discussed how prior authorizations for scans would take weeks, causing her significant anxiety.

“You know, if my doctor put in a request for a scan, or a test, it might be two to three weeks before I could get in… You know, it was like being on pins and needles.”—P02

One participant's initial surgery was delayed by another medical complication that required specialized care. Her insurance, however, did not cover care at her local hospital. Because her insurance‐approved facility had limited availability, eventually her gynecologic oncologist had to step in and advocate for her to be seen.

### Communication with providers

The ways in which providers would explain the treatment process and be transparent about what to expect made participants feel respected and cared for during treatment, leading to greater satisfaction with their care. Some participants discussed how this contrasted with their prior experiences in health care in general or their experiences with rural oncology care.

“Everybody being so nice. It's been years… I wouldn't even go back to a doctor in 30 years because I didn't like the doctors. They were mean…they wouldn't talk to you.”—P04

Clear communication about EC and what to expect during treatment also helped to offset discomfort from the invasiveness of oncology treatment and conveyed a sense of confidence and expertise that was valued by patients. The vast majority of patients stated they felt comfortable asking providers to explain things they did not understand about their diagnosis or treatment and appreciated the care and comfort communicated by providers.

“If I did feel like [I did not understand something], I would ask them to elaborate on it and he didn't mind breaking it down for me.”—P16

A few participants described instances where information about treatment or treatment decisions was not communicated in a way that supported a relationship of mutual respect between providers and patients. For example, overly positive or optimistic approaches to communication were perceived to be infantilizing by one patient.

“I'm old enough to take bad news. So, if the doctors see something, feel good news doesn't feel good when there's, you know, the bull behind it.”—P02

### Trust in rural health care and personal experiences driving care decisions

When deciding where to receive treatment, patients’ choices were often informed by prior personal experiences, level of trust in proximal sources of cancer care, community beliefs, or biases. Participants shared that their lack of trust in the quality of care of smaller, rural hospitals or cancer services in their area was because they perceived those providers to be less experienced and knowledgeable or that rural health care services simply did not have the resources to provide specialty care, especially oncology care. The source of this mistrust varied in terms of whether it came from personal or community beliefs. For example, one participant expressed it was common knowledge to go to an urban area for specialty care.

“…you hear the horror stories behind this hospital…So, you know, and that's the bad thing about living in a smaller town, you hear all that. Yeah, and that's the reason, that's probably the only reason why they all go to a bigger hospital because, when it's serious… you need to go somewhere where they got more experience.”—P08

Several patients discussed the impact that their personal experiences with health care (or the experiences of immediate family members) had on their care decisions—particularly on where to receive care. One participant's experience with her father's death from cancer factored into her decision to seek care at a larger, urban hospital because she was told by other doctors at an urban cancer center that the rural providers had not administered chemotherapy correctly to her father. Another participant received her initial cancer treatment at her local hospital, but when faced with needing treatment for a recurrence, she perceived her local oncologist to be too inexperienced. She watched videos of several gynecologic oncologists at urban cancer centers before referring herself to the one she felt was the most personable and experienced.

However, not all patients lacked trust in their local health care. One patient received a referral from her gynecologist to a larger, more distantly located hospital but preferred to be seen at a hospital more proximal to her home because the hospital was known to her.

“I think it made all the difference, that you could go to a hospital that you knew and that you were familiar with and had a good reputation.”—P13

Another participant noted individuals in her rural area were not willing to seek out medical care outside of their community.

“…people are not willing to go outside of the area because they are so comfortable in the area, in the rural areas”—P22

## DISCUSSION

This study aimed to qualitatively understand the determinants of diagnosis and treatment of rural EC patients. Lack of patient and provider knowledge of EC symptoms, patient symptom normalization, and patient fear were major factors delaying time from symptom onset to EC diagnosis. When undergoing treatment, we observed a wide array of experiences with barriers to care. Some participants experienced few barriers or burdens in accessing care. However, it was not uncommon to hear from patients who experienced financial burdens and substantial travel burdens that hindered their access to high‐quality care. Additionally, prior health care experiences and beliefs about rural health care also influenced decisions about where to seek gynecologic cancer treatment. During both diagnosis and treatment, participants reported the importance of family members and their social network in supporting them and facilitating their access to care.

Interventions are needed to address knowledge gaps in EC symptoms in order to improve timely diagnosis. To reach rural providers, for patients with PMB, current guidelines recommend further workup because of the sensitivity in the diagnostic tests used for accurate detection of EC.^24^ Adherence to these guidelines is critical as rates of EC continue to rise.^1^, ^25^, ^26^ Potential solutions include partnering with Project Extension for Community Healthcare (ECHO), an intervention model that integrates education and care management by connecting health care specialists and rural providers through telemonitoring and videoconferencing. Project ECHO has been shown to improve rates of colorectal cancer screening, HbA1c among diabetic patients, and quality of life among geriatric patients.^27^, ^28^, ^29^ Recently, Project ECHO was paired with patient navigation to improve the health and well‐being of rural cancer survivors; this model enhances both the expertise and skill set of rural multidisciplinary health care provider teams while also linking rural cancer survivors to supportive care resources.^30^ Using this combined approach, Project ECHO could be a useful tool to improve EC outcomes across the cancer control continuum, including improving provider knowledge of EC symptoms and PMB guidelines, or integrating with patient navigation to address barriers such as patient fear and anxiety. Ensuring interventions to improve knowledge of symptom recognition for both patients and providers that are tailored to the specific needs and resources of rural areas will be critical for improving early detection of EC.^31^

We found that support from family and social networks was integral to rural patients’ care experiences during both diagnosis and treatment. Given the role of social support during diagnosis in urging participants to seek care for their symptoms, there is a need to expand community knowledge and awareness of EC symptoms. While there are several community‐based programs that have been created to improve breast cancer knowledge and education among rural and minoritized populations, there is only one program, to our knowledge, that has been developed to address EC education.^43^, ^44^, ^45^ Specifically, the Community Empowerment Partners for Endometrial Cancer (CEPs‐EC) is a peer education program designed to increase Black women's knowledge of and confidence in discussing AUB, menopause, and EC with their peers and in clinical settings. CEPs‐EC has been shown to be effective in increasing participants’ EC knowledge and social confidence.^44^ Similar community‐driven programs to improve EC knowledge need to be developed for, tailored to, and diffused among rural populations.

During EC treatment, interventions to address travel burdens and costs are needed. Recent literature shows that more than half (56%) of EC patients travel for surgical care, the cornerstone of treatment.^32^ The extensive travel required of rural EC patients to access high‐quality cancer care can exacerbate patients’ financial burden with approximately half (47% to 51%) of gynecologic oncology patients experiencing financial toxicity.^33^, ^34^, ^35^, ^36^, ^37^, ^38^ One approach to lowering expenses is through telemedicine, which has been shown to reduce patient costs by over $145 per visit.^39^ In particular, gynecologic oncology patients have been shown to be willing to use telemedicine.^40^ Of course, since treatment must still be delivered in person, transportation and financial resources need to continue to be offered to patients (as well as their caregivers).

Notably, few participants in our sample discussed being aware of transportation or financial assistance. This may be due, in part, to the overwhelming emotions patients may feel when receiving a diagnosis and treatment plan for cancer; these heightened emotions, in addition to being unaware of the high costs until treatment has been completed, may limit patients’ ability to think about resources and assistance they might need while going through such a life‐altering experience. Regardless, the lack of awareness of the availability of financial assistance resources (for both travel and health care costs) demonstrates a need to improve comprehensive screening for supportive resources or to modify how and when such resources are discussed with and offered to patients. In recent studies evaluating the implementation of screening tools among gynecologic oncologic patients, transportation assistance was the most frequent unmet need; almost one third of patients needed transportation support (32%), followed by general financial assistance (20%).^41^, ^42^ A comprehensive strategy that assesses the challenges of travel and costs across the treatment experience (as needs change over time) is essential to ensure fair access to quality cancer care for rural EC patients.

### Limitations

This study has several limitations. While all the patients were recruited from rural counties, they were primarily from the central and eastern regions of a single state (Figure 1). Patients living in rural areas in other areas of the United States (such as the rural northeast) may face different barriers to care. For example, one previous study from New Hampshire found that being diagnosed during the winter led to rural breast cancer patients forgoing radiation treatment.^46^ Nevertheless, within North Carolina, our study was able to represent nearly a quarter of rural areas in the state and the experiences of patients from rural areas with higher populations of Black residents (Table 1; Figure 1).

Second, there is also some selection bias in our sample. While all participants recruited were from rural counties, they all received care (either in its entirety or in part) at a high‐volume cancer care facility. Additionally, rural patients are at greater risk of not receiving care from a gynecologic oncologist, but participants in our study were all patients who at least had an initial consult with, if not all their care directed by, a gynecologic oncologist. Thus, despite numerous barriers to care identified by our participants, the experiences of participants in this study may not adequately reflect the experiences of patients who were never seen by a gynecologic oncologist. Our participants expressed positive attitudes about their experience with their gynecologic oncologists. Patients who may have experienced greater barriers to care or negative experiences with providers may not have been as inclined or have capacity to participate in a research study.

Finally, we interviewed the caregiver of one patient who was unable to conduct an interview about the patient's experiences accessing care. A caregiver's perception of barriers to care, particularly as it relates to symptom recognition prior to diagnosis and interpersonal experiences with providers, may be a limited representation of the patient experience. However, the caregiver was heavily involved in the patient's treatment experience, and they were able to provide a valuable representation of the logistic barriers that were faced (particularly around cost and transportation) during diagnosis and treatment; therefore, we included the caregiver interview in the analysis.

## CONCLUSIONS

This study highlights the complex and multifaceted barriers rural patients face in the diagnosis and treatment of EC. From limited knowledge of EC symptoms during diagnosis to financial hardship and travel burdens during treatment, rural patients encounter numerous obstacles that may hinder the receipt of timely, high‐quality care. Our findings emphasize the need for targeted interventions that improve symptom awareness among both patients and providers during diagnosis. During treatment, there is a clear need to expand supportive services such as patient navigation and telemedicine and to improve communication about available financial and transportation assistance. Furthermore, strengthening community‐based education and leveraging the support of family and social networks may play a critical role in improving both early detection and treatment.

## CONFLICT OF INTEREST STATEMENT

L.P.S. has received salary support paid to her institution for unrelated work from AstraZeneca. S.B.W. has received salary support paid to her institution for unrelated work from Pfizer Foundation/NCCN and AstraZeneca. The other authors declare no conflicts of interest.

## Supporting information



Supporting Information

## Data Availability

The data used in this study cannot be disclosed due to the privacy concerns of the participants. Given the personal and qualitative nature of the interviews and experiences, we have also removed identifying details from individual transcripts that could potentially compromise confidentiality.
