# Supplementary material for: Rural patients’ experiences with diagnosis and treatment of endometrial cancer
Source: J Rural Health. 2025 Aug 22;41(3):e70065. doi: 10.1111/jrh.70065 (PMC12372867; doi:10.1111/jrh.70065)
Supplement: Supplementary file 1 — Supporting Information [file JRH-41-0-s001.docx]

**Rural Patients’ Experiences with Diagnosis and Treatment of Endometrial Cancer**

**Supplementary Material: Interview Guide**

**Diagnosis**

1. Tell me more about your experience getting diagnosed with endometrial cancer.
   1. Prompts/Probes:
      1. How and when did you know something was wrong?
      2. What was the bleeding like?
2. What provider or who did you go see first about it? (e.g., primary care, OBGYN, emergency room).
3. What did your doctor tell you about your symptoms/ what was wrong
4. What stage of endometrial cancer were you diagnosed with?
5. How long was it before you saw a gynecologic oncologist?
6. Tell me about something that was challenging during your experience being diagnosed with endometrial cancer.
7. Who or what supported you in the process of getting diagnosed?

**Treatment**

1. Tell me about your experience receiving treatment for endometrial cancer.
   1. Prompts:
      1. What treatments did you receive to treat your endometrial cancer?
      2. Where did you receive each of your treatments?
      3. What was the location of your treatment facility(ies)?
2. *(If patient underwent treatments other than surgery)* What were some of the factors that you considered when deciding whether to do [chemotherapy & radiation OR just chemotherapy OR just radiation]?
3. What types of issues or problems did you encounter while receiving treatment?
   1. Did you ever feel like you did not understand your diagnosis or what was happening during your treatment?
   2. Was it ever difficult to communicate with your providers or other people that worked at the clinics or hospitals where you were treated?
4. Can you tell me about some things you experienced during your treatment that were difficult?
5. What was your experience navigating the cost of treatment and insurance coverage?
6. Can you describe any lasting financial impacts resulting from your treatment?
7. Were you given any [additional] resources to help you while receiving treatment?
   1. Follow-up: How helpful was that resource?
8. How have your family and/or community been involved in supporting you during your treatment?
9. Can you tell me about any delays you experienced during your cancer treatment?
10. Any instances where you avoided seeking medical care during treatment even when you felt like you needed to?
11. Tell me about managing your symptoms during treatment (like pain, fatigue, or stomach problems).
12. Were there times where you felt like you couldn’t ask for help managing your symptoms?
13. Do you have any suggestions for improving the treatment process for endometrial cancer based on your experience?
    1. What advice would you give to future patients going through endometrial cancer treatment?
    2. What advice would you give providers in treating patients with endometrial cancer?
14. How satisfied were you with the care you received during your cancer treatment?
15. How did COVID-19 impact your treatment experience?
16. Is there anything else you would like to share related to your experience receiving treatment for endometrial cancer?
